# The Role of Glia in Wilson’s Disease: Clinical, Neuroimaging, Neuropathological and Molecular Perspectives

**DOI:** 10.3390/ijms25147545

**Published:** 2024-07-09

**Authors:** Grażyna Gromadzka, Anna Wilkaniec, Beata Tarnacka, Krzysztof Hadrian, Maria Bendykowska, Adam Przybyłkowski, Tomasz Litwin

**Affiliations:** 1 Department of Biomedical Sciences, Faculty of Medicine, Collegium Medicum, Cardinal Stefan Wyszynski University, Wóycickiego 1/3, 01-938 Warsaw, Poland; 2 Department of Cellular Signalling, Mossakowski Medical Research Centre, Polish Academy of Sciences, 5 Pawińskiego St., 02-106 Warsaw, Poland; 3 Department of Rehabilitation, Medical University of Warsaw, Spartańska 1, 02-637 Warsaw, Poland; 4 Department of Gastroenterology and Internal Medicine, Medical University of Warsaw, Banacha 1a, 02-097 Warsaw, Polandaprzybylkowski@wum.edu.pl (A.P.); 5 Students Scientific Association “Immunis”, Cardinal Stefan Wyszynski University, Dewajtis 5, 01-815 Warsaw, Poland; 6 Second Department of Neurology, Institute of Psychiatry and Neurology, Sobieskiego 9, 02-957 Warsaw, Poland

**Keywords:** Wilson’s disease, glia, neurogeneration, neuroprotection, neuropathology, astrocytes, copper, iron, cuproptosis, ferroptosis

## Abstract

Wilson’s disease (WD) is inherited in an autosomal recessive manner and is caused by pathogenic variants of the *ATP7B* gene, which are responsible for impaired copper transport in the cell, inhibition of copper binding to apoceruloplasmin, and biliary excretion. This leads to the accumulation of copper in the tissues. Copper accumulation in the CNS leads to the neurological and psychiatric symptoms of WD. Abnormalities of copper metabolism in WD are associated with impaired iron metabolism. Both of these elements are redox active and may contribute to neuropathology. It has long been assumed that among parenchymal cells, astrocytes have the greatest impact on copper and iron homeostasis in the brain. Capillary endothelial cells are separated from the neuropil by astrocyte terminal legs, putting astrocytes in an ideal position to regulate the transport of iron and copper to other brain cells and protect them if metals breach the blood–brain barrier. Astrocytes are responsible for, among other things, maintaining extracellular ion homeostasis, modulating synaptic transmission and plasticity, obtaining metabolites, and protecting the brain against oxidative stress and toxins. However, excess copper and/or iron causes an increase in the number of astrocytes and their morphological changes observed in neuropathological studies, as well as a loss of the copper/iron storage function leading to macromolecule peroxidation and neuronal loss through apoptosis, autophagy, or cuproptosis/ferroptosis. The molecular mechanisms explaining the possible role of glia in copper- and iron-induced neurodegeneration in WD are largely understood from studies of neuropathology in Parkinson’s disease and Alzheimer’s disease. Understanding the mechanisms of glial involvement in neuroprotection/neurotoxicity is important for explaining the pathomechanisms of neuronal death in WD and, in the future, perhaps for developing more effective diagnostic/treatment methods.

## 1. Introduction

Wilson’s disease (WD) (Online Mendelian Inheritance in Men, OMIM #277900) was first described in 1912 by the British neurologist Samuel Alexander Kinnier Wilson as “progressive lentihepatic degeneration” [1]. Over the following decades, the importance of copper in the pathogenesis of the disease was determined [2,3].

WD belongs to the group of genetically determined metabolic disorders. It is inherited in an autosomal recessive manner. In 1993, it was discovered that mutations in the *ATP7B* gene located on chromosome 13 (13q14.3) were responsible for the disease. This gene encodes ATPase7B [OMIM *606882]—an enzyme located mainly in the membranes of the structures of the Golgi apparatus [4,5].

Over 950 mutations of the *ATP7B* gene have been described [6].

The physiological function of ATPase7B is the active transport of copper in cells [4]. Changes in the structure of this protein and/or reduction in its enzymatic activity are associated with impaired intracellular copper transport, inhibition of copper binding to apoceruloplasmin in liver cells, and impaired copper excretion via the intestinal bile [7]. Impaired intracellular copper transport is the cause of this element’s accumulation in hepatocytes. The consequence of excessive copper accumulation is a toxic effect with progressive damage to hepatocytes. This is related to the release of free copper ions into the bloodstream, which can accumulate in various organs, including in the brain. Excessive accumulation of copper within the central nervous system (CNS) leads to neurodegeneration [8,9]. Research results indicate that glia play an important role in neurodegeneration in WD. Impairments and disorders of astroglial function, giving the picture of “primary gliopathy”, are the leading morphological feature of the neuropsychiatric syndromes of WD [10].

## 2. Wilson’s Disease—Neuropsychiatric Symptoms

Depending on the dominant symptoms, different clinical forms of WD are distinguished: hepatic (approx. 45% of patients), neurological (approx. 35% of patients) and psychiatric (approx. 10% of patients). Rarely, the first symptom of the disease is hemolytic anemia, Fanconi syndrome, pancreatitis, hypothyroidism and parathyroidism, or cardiomyopathy [8,11,12,13].

In patients with hepatic cirrhosis, morphological alterations primarily affect astrocytes and microglia within the brain. In WD, hepatic failure can cause hepatic encephalopathy symptoms resembling parkinsonian signs as bradykinesia, rigidity or tremors; asterixis (flapping motions of outstretched, dorsiflexed hands), ataxia, slurred speech, hyperreflexia, a positive Babinski sign, multifocal myoclonus and nystagmus. Alterations in sleep patterns or cognitive capacity may manifest also in hepatic encephalopathy and attention deficits and impaired short-term memory. The age of onset of symptoms is variable, with cases of the disease being diagnosed from the age of 2 to 70 and over [14,15]. However, symptoms are usually not observed before the age of 3, which is most likely due to the storage capacity of the liver. Neurological symptoms occur later compared to liver symptoms, most often as one of the first symptoms, in the second or third decade of life. Initially, they are very subtle and increase as the disease progresses. They reflect damage to the subcortical nuclei visible in neuroimaging studies [11,12,13]. Some of these symptoms include tremor, Parkinson-like symptoms such as hypomimia and salivation, ataxia, and dystonia. Chorea and athetosis occur in approximately 10% of WD cases. Dysarthria in patients with WD can present as mixed dysarthria with various spastic, ataxic, and dystonic features [16].

Psychiatric symptoms are common in adult patients with WD. They are present in about 25 percent of patients at the time of initial diagnosis. The main symptoms are behavioral disorders, mood disorders (including mania, depression), cognitive deficits (mainly disorders of executive and visuospatial functions). Cognitive deficits (slow processing speed and mild impairments in working memory, attention, and abstract thinking) are likely due to changes in corticostriatal pathways. They are correlated with the global severity of magnetic resonance imaging (MRI) abnormalities, such as hyperintense lesions, metal accumulation in the basal ganglia, and subcortical and cortical atrophy. Other psychiatric disorders such as catatonia, anorexia nervosa, bulimia nervosa, obsessive–compulsive disorder and attention deficit hyperactivity disorder (ADHD) have also been reported in WD [12,16]. Sleep disturbances have also been reported in WD patients. They are most likely caused by accumulation of copper and subsequent degeneration of the nervous system of the brainstem [12,16].

## 3. CNS Pathology in WD—Neuroimaging Studies

**Figure 1:**
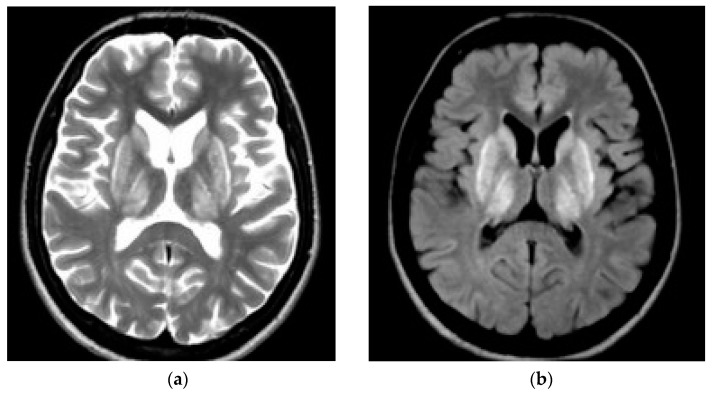
Symmetrical changes visualized in putamen, caudate, and thalamus with bright claustrum sign in brain MRI in T2-weighted sequences (**a**) and FLAIR sequences (**b**) (source: own material from 2nd Department of Neurology, Institute Psychiatry and Neurology, Warsaw, Poland).

In WD, neuroimaging studies show pathology mainly in the basal ganglia of the brain, and sometimes in the brainstem (also very rarely due to changes in the white matter in magnetic resonance imaging (MRI)). Initially, in the early 1980s, before MRI was widely introduced, these changes were visualized (mainly in patients with neurological BD) as hypotensive changes in the basal ganglia (gray matter nuclei), which mainly reflect necrosis (advanced disease) with brain atrophy (cortical, cerebral, and subcortical) [17,18,19]. Lesions characteristic of brain MRI lesions in people with diabetes include symmetric, hyperintense (or mixed) lesions on T2-weighted and FLAIR (Fluid-Attenuated Inversion Recovery) sequences located in the putamen, globus pallidi, caudate nuclei, thalamus, and pons (see: Figure 1). Other structures such as the corticospinal tracts and midbrain may also be affected. Additionally, changes in the white matter may occur in up to 20% of patients with bipolar disorder (hypocupemia associated with anticopper treatment is suggested as the pathomechanism). In advanced stages of the disease, it may be visible as hypointense on T1-weighted sequences (reflecting necrosis). Some cirrhotic patients additionally present with non-WD-specific but liver disease-specific symmetric T1 hyperintense lesions in the basal ganglia, which probably reflect manganese accumulation. Moreover, most patients with long-term WD show brain atrophy, which is particularly accelerated in neurological WD, although also detectable in hepatic WD, which highlights the copper toxicity in the brain and neurodegeneration in the course of WD [20,21,22,23]. It should be mentioned that WD-specific changes in brain MRI occur in almost 100% of WD patients with neurological diseases, in 40% of WD patients with hepatic symptoms, and even in 20% of clinically asymptomatic patients [21]. For this reason, brain MRI changes typical of bipolar disorder are even included in the Leipzig algorithm—for the diagnosis of bipolar disorder (2 points) [24]. Moreover, based on the pathomechanism of WD, brain MRI lesions can be divided based on the semi-quantitative brain MRI scale proposed by Dusk et al. [25] as acute, potentially reversible (“acute toxicity”—visualized as hyperintense in T2 and FLAIR sequences) and chronic irreversible (“chronic damage”—visualized in T2/T2*/SWI sequences and with brain atrophy assessed in T1 sequences). Acute changes pathologically reflect edema, demyelination, chronic irreversible changes reflect necrosis, atrophy, and iron accumulation (mainly due to the influx of macrophages into necrotic lesions). The proposed MRI brain damage scale correlates very well with the clinical neurological severity of the disease assessed in the Unified WD Rating Scale (UWDRS), as well as with serum biomarkers of neurological damage (e.g., neurofilament light chain) [20,24,25]. This further confirms the reliability of brain MRI in monitoring WD and encourages the use of this scale in clinical trials.

Initially, the brain atrophy observed in patients with WD was subjectively described by a neuroradiologist. Currently, volumetric studies analyzing brain atrophy are performed using objective tools—programs such as SIENAX, Free-surfer or voxel, or surface morphometry, which have shown that gray matter volume is particularly important in central structures (basal ganglia, thalamus, brainstem, and cerebellum) and orbitofrontal cortex), but especially, the volume of the putamen is related to the severity of the neurological disease [23,26,27]. Moreover, when assessing longitudinal (baseline and 12-month follow-up) cerebral atrophy, the percentage change in ventricular volume (PVVC) in neurological patients is almost 10-fold higher than in other WD patients (median 5.49% vs. 0.5%), and in patients with the neurological form of WD it was even 16.72%. These observations indicate direct and indirect copper toxicity in the brain and that initially, regardless of clinical symptoms, the neurodegeneration process in WD seriously affects brain volume [26]. Another interesting brain MRI finding is the so-called “pathognomonic” neuroradiological manifestation of WD. Since in neurology there are several neuroradiological signs suggesting various disorders (e.g., “butterfly sign”—glioma, tiger eye sign—a form of neurodegeneration with accumulation of iron in the brain, etc.), several neuroradiological features in brain MRI have also been proposed in WD: (1) “facial giant panda” in the midbrain; (2) “miniature panda” in the bridge, (3) “light claustrum”—claustrum; (4) “split thalamus”—thalamus, and (5) “whorl” symptom—putamen [28,29]. These changes occur in almost 43% of neurological forms of WD, but they are not pathognomonic for WD, as they may occur in other diseases. However, due to their typical presentation and high prevalence rate (especially the “giant panda” pattern observed in 27% of patients with neurological WD), they should be taken into account in the differential diagnosis of extrapyramidal disorders [29].

## 4. Glia in CNS Pathology in WD

The role of glia in CNS pathology in WD should be considered, taking into account the importance of not only copper but also iron in the pathogenesis of neuronal damage.

There are several studies documenting abnormalities in iron metabolism, as well as the accumulation of this element in the liver and in the brains of patients with WD [30,31,32,33,34,35]. Impairment of iron metabolism results mainly from disturbances in the synthesis/enzymatic function of ceruloplasmin (CPN), which, due to disturbances in copper metabolism, is not transformed into a mature, fully active holoCPN molecule, the presence of which is fundamentally important for proper iron metabolism.

The first data about brain iron accumulation in WD come from neuroimaging studies (brain MRI), which showed reduced signal intensity in basal ganglia in T2-weighted images, similar to disorders with brain iron accumulation (neurodegeneration with brain iron accumulation; NBIA) [36,37,38,39,40].

Also, indirect neuroimaging assessment with new brain MRI techniques like susceptibility weighted imaging (SWI), gradient echo (T2*), relaxometry (R2*) and with postprocessing analyses with quantitative susceptibility mapping (QSM) suggested that iron may accumulate in WD, and it could correlate with the severity of WD as well as with neurological deterioration [35,36,37,38,39,40]. Based on these indirect data, the neuropathological studies directly confirming this hypothesis were performed [41,42].

Dusek et al. analyzing nine brains of WD patients and six controls, documented that changes suggested in brain MRI as iron accumulation in WD were equal to iron accumulation (immunohistochemistry and iron concentrations analysis with flame atomic absorption spectroscopy), especially in putamen, nucleus caudatus, and pons (tegmentum). In immunohistochemical studies, iron was detected mainly in macrophages, and the degree of tissue destruction resulting from WD correlated with the presence of iron-containing macrophages [43].

The iron accumulation in the brains of WD patients is regarded as a secondary phenomenon to the phagocytic cells’ influx to the brain lesions caused by copper-induced tissue destruction [43].

Both copper and iron are redox-active metals and their transformations are related to oxidative stress. Their accumulation in the tissue can also cause cellular damage through other mechanisms, including inflammation, mitochondrial toxicity, cell membrane damage, DNA cross-linking, and inhibition of multiple enzymes [3].

Glia have been shown to play an important role in cerebral metal metabolism and homeostasis and may be important in protecting neurons from toxicity associated with excessive copper and iron accumulation in WD.

### 4.1. Glia in Brain Copper Homeostasis

Copper homeostasis is crucial for the proper functioning of the nervous system. Like other metals such as iron, copper is highly concentrated in the CNS, making the brain one of the most important copper-dependent organs [44]. Copper is generally twice as abundant in gray matter than white matter [45]. In humans, the highest concentrations of copper are observed in the hippocampus, mainly in the granule and pyramidal cell layers of the dentate gyrus and CA1 area [46]. Significant levels of copper are also found in the in the locus coeruleus, substantia nigra [47], cerebellum, hypothalamus, olfactory bulb, and cortex [44,48]. Copper in brain serves as a cofactor for many enzymes (called cuproenzymes) with important physiological functions in the brain, including the dopamine β-hydroxylase like monooxygenase, which catalyzes norepinephrine synthesis [49]; cytochrome c oxidase in mitochondria [50]; amine oxidases, which synthesize neurotransmitters; Cu/Zn superoxide dismutase (Cu-Zn SOD), which catalyzes the process of dismutation of superoxide radicals to oxygen and hydrogen peroxide [51]; or tyrosinase, which forms melanin [52].

Copper enters the brain through the blood–brain and blood–cerebrospinal fluid (CSF) barriers, facilitated by copper transporter 1 (CTR1) [53] and the P-type ATPase, ATP7A (also known as Menkes’ protein) [54]. These transporters are expressed in the endothelial cells of the blood–brain barrier and the epithelial cells of the choroid plexus at the blood–CSF interface [55,56].

It is suggested that astrocytes might play a role in further regulating copper transport from systemic fluids, potentially acting as an additional blockade. Indeed, among the parenchymal cells, astrocytes seem to play a crucial role in regulating copper homeostasis in the brain [57]. Studies have shown that astrocytes efficiently absorb copper from the culture media [58]. They manage the uptake, storage, and release of copper to neurons, ensuring the balance required for proper neuronal function.

Astrocytes uptake copper into the cytoplasm by the CTR1 and to a lower extent through divalent metal transporter 1 (DMT1) [59,60,61], carrying preferentially monovalent/divalent copper, respectively [62]. Therefore, the delivery of Cu+ for cellular uptake relies on the reduction of extracellular Cu^2+^, a process that can be facilitated by small molecule reducing agents like ascorbate, which is released by astrocytes [63]. Moreover, an ecto-cuprireductase on the plasma membrane of astrocytes is also involved in reducing extracellular copper, enabling cultured astrocytes to accumulate copper rapidly upon Cu^2+^ administration [64,65].

**Figure 2:**
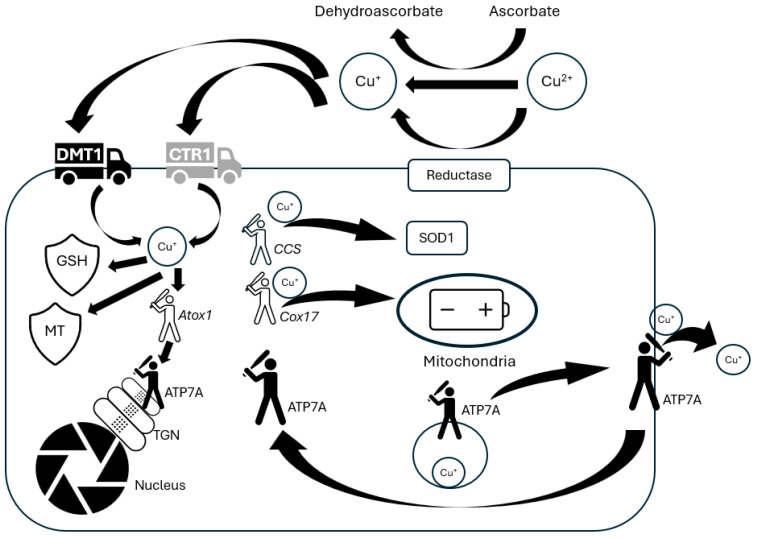
Copper transport paths in astrocytes. Copper can be taken up into astrocytes by copper transporter receptor 1 (Ctrl) and probably also by divalent metal transporter 1 (DMT1). These transporters have been reported to prefer Cu^+^ as a substrate. Astrocyte ecto-cuprireductase and/or extracellular ascorbate may reduce Cu^2+^ for uptake. Accumulated copper is sequestered by glutathione (GSH) in astrocytes or stored in metallothioneins (MT). In addition, copper is shuttled to its specific cellular targets by the copper chaperones: copper chaperone for superoxide dismutase (CCS) to superoxide dismutase 1 (SOD1), Coxl7 to cytochrome c oxidase, and antioxidant protein 1 (Atox l) to ATP-ase 7A (ATP7A). ATP7A transports copper to the trans-Golgi network (TGN) to then bind to copper-dependent enzymes such as ceruloplasmin (CPN). When cellular copper levels rise above a certain threshold, ATP7A is reversibly translocated to the plasma membrane via vesicles. ATP7A brings copper into vesicles for release by fusion with the plasma membrane and/or exports copper directly.

Once imported to astrocyte, Cu^+^ is directed to various copper storage sites, like glutathione (GSH) and metallothioneins (MTs) [66] (see Figure 2).

**Figure 3:**
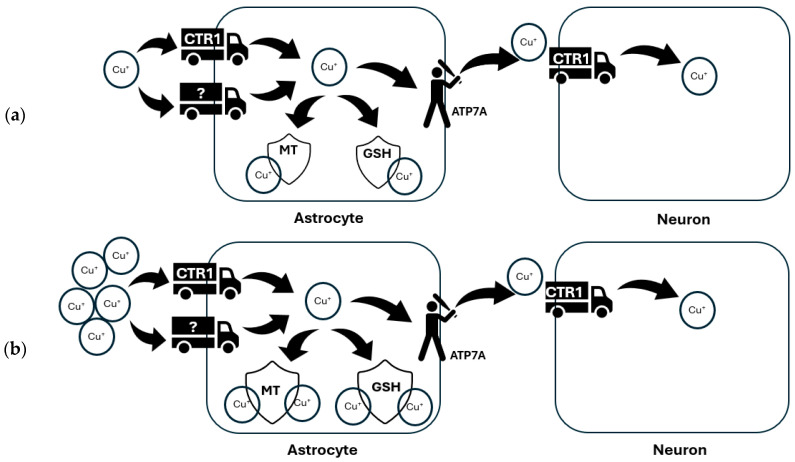
Copper transport paths in astrocytes. A proposed model for astrocytic copper delivery to neurons. (**a**) In the normal brain, astrocytes efficiently take up copper via Ctr1 and other unidentified transporters. Excess copper is sequestered by glutathione (GSH) in astrocytes or stored in metallothioneins (MT), and copper is released via ATP7A to supply neurons with copper. (**b**) Under conditions of copper overload, excess copper is efficiently absorbed by astrocytes and stored in MT or as a GSH complex to prevent copper-induced neurotoxicity.

ATP7A mediates the release of copper from astrocytes to supply neurons with this essential metal [67] (see: Figure 3a), thus impaired copper supply from astrocytes induced by ATPase mutation may contribute to the neuronal copper deficiency in Menkes diseases [68,69,70]. On the other hand, the excess of copper in the brain, which is observed in aging [71] and accompanying various CNS disorders like WD or Parkinson’s disease (PD) [72] is sufficiently buffered by astrocytes, accompanied by their proliferation, acceleration in astrocytic glucose consumption and lactate release [73], the elevation of MTs [61,74] and GSH production [66] to increase copper storage ability (see: Figure 3b). Apart from the maintenance of copper balance, astrocytes are also essential for neuroprotection from oxidative stress induced by this redox-active ion by secreting GSH, which can reduce copper-mediated GSH depletion in neurons [75], or by the release of pyruvate which prevents the autooxidation of cysteine induced by copper [76].

### 4.2. Glia in Copper-Induced Neuronal Degeneration: Lesson from Alzheimer’s Disease

As copper continues to accumulate in the brain, it can eventually surpass the buffering capacity of astrocytes, leading to copper overload in neuronal cells and their subsequent death through the mechanism of cuproptosis. Cuproptosis is triggered by the accumulation of copper in mitochondria that drives the aggregation of lipoylated dihydrolipoamide S-acetyltransferase (DLAT), which is associated with mitochondrial tricarboxylic acid (TCA) cycle, leading to proteotoxic stress, and ultimately cell death [77]. Cuproptosis primarily occurs in cells that produce energy and mainly use oxidative phosphorylation (OXPHOS) as the main metabolic pathway [78], thus neurons are especially vulnerable to this type of death. Therefore, it seems plausible to search the new therapeutic approaches that facilitate both mechanisms of astrocytes-mediated neuroprotection against copper-induced neurotoxicity in CNS diseases.

Copper also plays an essential role in the activation of microglia and this process has been most extensively described in Alzheimer’s disease (AD). In this disorder, hyperactivated microglia surrounding Aβ plaques upregulate the expression of ATP7A, which may favor overall copper uptake by augmenting CTR1 importer expression [79]. Also, proinflammatory agents, which are released from glial cells in response to Aβ, were shown to increase the expression of ATP7A, CTR1, and the following elevation of Cu uptake by microglial cells [79]. The intracellular sequestration of Cu^2+^ has been reported to activate the neuroprotective phenotype of microglia, which may further inhibit copper-mediated Aβ plaque formation [80]. Also, the redox state of copper influences the microglial phenotype, as Cu^+^ was demonstrated to polarize microglia from a proinflammatory M1 phenotype to an anti-inflammatory M2 phenotype by inhibiting NO production and regulating S-nitrosothiol signaling [81,82]. Cu^+^ decreases Aβ aggregation and ROS production, suggesting lower neurotoxicity compared to Cu^2+^, which forms neurotoxic complexes with Aβ [83,84]. The oxidized copper triggers activation of the NF-κB signaling pathway and increases the release of inflammatory factors such as nitric oxide (NO) and the tumor necrosis factor-α (TNF-α) [85]. These proinflammatory factors can work together to induce morphological changes in microglia, transitioning them from a resting to an active state. This activation ultimately leads to microglia-mediated neuroinflammation and neuronal damage [80,86]. Some studies suggest that copper may impair microglial clearance of Aβ by affecting the mTOR axis, inhibiting lysosomal biogenesis and autophagic flux, and decreasing the expression of lysosomal biogenesis and Aβ clearance genes (LAMP 1/2, Cat B) [87]. Moreover, Cu forms complexes with Aβ peptides, which results in the reduction in lipoprotein receptor-related protein 1 (LRP1), which is related to Aβ clearance in the brain [88], thus copper in excess may also reduce the brain’s physiological ability to remove Aβ peptides [85]. Also, elevated CPN levels in AD patients can enhance proinflammatory microglial activation by upregulating NO release and proinflammatory gene expression [89]. Recent data point out that excess copper intake may shift microglial phenotypes towards hyperactivation by upregulating the expression of genes related to neurodegeneration. This was suggested to be responsible for the acceleration of cognitive decline occurring in AD pathology [90]. On the other hand, a lack of copper can also lead to aberrant microglial activation contributing to neurodegenerative diseases [91].

In summary, the intricate balance of copper metabolism maintained by astrocytes and microglia is vital for neuronal health. Disruptions in this balance, due to genetic mutations, environmental factors, or disease states, can lead to neurodegenerative processes. Understanding the mechanisms by which glial cells regulate copper and their interaction with neurons is crucial for developing therapeutic strategies for CNS diseases.

### 4.3. Glia in Brain Iron Homeostasis

Iron is essential for various cellular processes in the brain, such as neurotransmitter synthesis, myelination, and mitochondrial function. Thus, maintaining an adequate supply of iron is necessary to sustain the brain’s high-energy demands [92,93]. As a result, iron ranks as the most abundant metal in the brain [94], functioning as a crucial co-factor involved in various processes within CNS such as redox homeostasis, mitochondrial respiration, processes of myelination and remyelination, neurotransmitter synthesis, and metabolism [95,96]. However, excessive intracellular iron accumulation can lead to neurotoxicity [97]. Therefore, precise control of brain iron metabolism is essential to uphold normal physiological brain function. Growing evidence shows that glial cells have a vital role in maintaining iron balance within the CNS, by regulating iron uptake, storage, and release, ensuring its availability to neurons while preventing iron overload, which can lead to oxidative stress and neuronal damage. Therefore, any disruption in the function of glial cells could result in dysfunction of iron regulation and subsequent neuronal degeneration.

**Figure 4:**
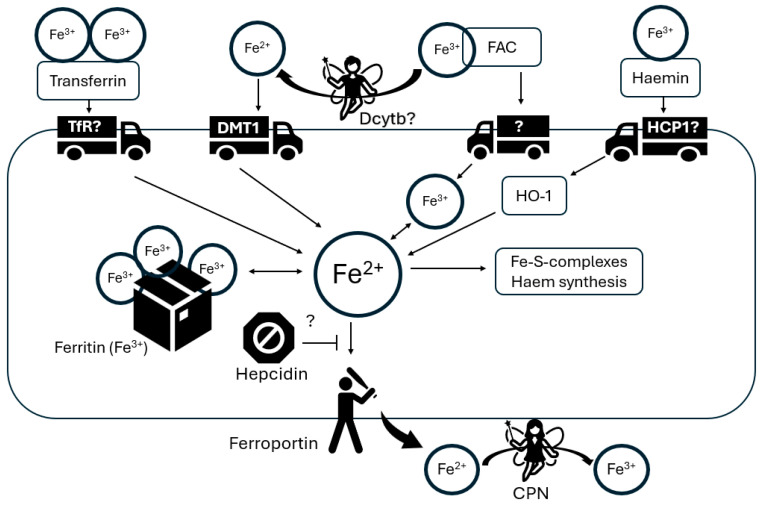
Iron transport paths in astrocytes.

Astrocytes, the predominant glial cells in the brain, are strategically positioned to obtain nutrients from the bloodstream [98] and are involved in iron uptake from the blood–brain barrier and its distribution to neurons [99]. Their end-feet processes establish close associations with the abluminal side of brain capillary endothelial cells (BCECs), which likely serve as the primary gate for iron entry into brain tissue through the transferrin (Tf)/transferrin receptor (TfR1) pathway [100,101]. Nonetheless, the mechanisms governing iron uptake and release in astrocytes during postnatal brain development are not fully investigated and remain relevant questions in the field. In the physiological conditions in vivo, astrocytes were shown to be devoid of TfR1, suggesting that they take up iron by other mechanisms that do not involve the transferrin receptor [102]. The likely mechanisms responsible for iron uptake by astrocytes include DMT1 [103], zinc transporter Zip14 [104] or resident transient receptor potential channel (TRPC) [105]. Among those, DMT1, which is mainly expressed in the astrocytic processes related to the vascular endothelial cells [106,107], seems to play a major role in iron absorption in astrocytes. Moreover, this transporter is likely involved in the astrocyte-mediated iron redistribution within the brain [106]. The ferroportin (FPN) and CPN that represent the main pathway for iron efflux are both highly expressed in astrocytic cell membranes, playing an important role in iron mobilization from these cells into the extracellular brain space [108,109,110] (see: Figure 4). Notably, astrocytes seem to be mostly involved in iron trafficking, not in its accumulation, because within the glial cells, they have the lowest metabolic prerequisite for iron [111] and have the least iron storage capacity. Astrocytes have relatively low levels of ferritin, particularly in the human striatum, which is known for its high iron content [112]. Nevertheless, brain aging results in an increase in the population of iron-positive astrocytes [113], where iron is stored not only in ferritin but also in astrocytic mitochondria [114].

Microglia, the resident immune cells of the CNS, also actively participate in iron metabolism to support neuronal function and protect against oxidative stress. There are several proteins involved in iron metabolism in microglial cells, including DMT1, TfR, ferritin, and ferroportin [115,116,117]. Thus, microglial cells can internalize both NTBI and Tf iron through different pathways of uptake [118]. After the release from Tf in the acidic environment of the endosome, iron is translocated into the cytoplasm by DMT1 or other transporters [119]. In addition to oligodendrocytes, microglia appear to have a high ferritin content, which mostly consists of L-chains [120,121], and are the most efficient in NTBI accumulation among all glial cells [122]. Microglial cells regulate their iron levels through iron regulatory proteins (IRP1 and IRP2) and the iron-responsive element (IRE) signaling pathway, which controls the expression of proteins involved in iron metabolism [123]. When intracellular iron levels are low, IRPs bind to iron-responsive elements (IREs) in the mRNA of transferrin receptor and ferritin, leading to increased expression of those proteins, and promoting iron uptake and storage [124].

Transferrin-bound iron can be taken up by transferrin receptor (TfR)-mediated endocytosis into astrocytes. In addition, astrocytes express divalent metal transporter 1 (DMT1), which mediates Fe^2+^ uptake. Because astrocytes can accumulate iron from extracellular ferric ammonium citrate (FAC), they must either have an iron transporter or have iron reductase activity on their outer cell membrane. Astrocytes express iron reductase Dcytb in vitro, but its presence in the brain has not yet been confirmed. Astrocytes can also remove iron from hemin, possibly via the heme transporter (HCP1), although the transporter involved has not yet been identified. Heme oxygenase-1 (HO-1) reduces intracellular hemoferrin. The pool of soluble iron in astrocytes serves the synthesis of heme and iron–sulfur clusters. Excess iron is stored in ferritin. Fe^2+^ is exported from astrocytes by ferroportin. This process can be regulated in astrocytes by hepcidin, which binds to ferroportin and induces its degradation. Exported Fe^2+^ is immediately oxidized to Fe^3+^ by CPN. The symbol “?” indicates that these components have not yet been shown to be expressed by astrocytes or have not yet been observed in astrocytes in vivo.

### 4.4. The Lack of Functional Ceruloplasmin—Role in Neurodegeneration in WD

In WD, due to disturbances in copper metabolism, the formation of the mature, active form of CPN is reduced [125]. The main source of CPN in the brain is believed to be astrocytes, which produce a form of CPN bound to glycosylphosphatidylinositol (GPI). This form of CPN plays a role in iron metabolism in the brain. It has not yet been established how CPN prevents iron accumulation in the brain. As was mentioned above, iron uptake in the brain may occur through transferrin receptors located on the endothelial cells of cerebral vessels. Other mechanisms involving the DMT1 iron transporter also play an important role. Perhaps GPI-CPN plays a role in reducing the amount of iron that is transferred by DMT1, which is specific for Fe^2+^ ions. CPN probably also plays a role in mediating the excretion of iron from cells via ferroportin by oxidation of Fe^2+^ to Fe^3+^. Due to its ferroxidase properties, CPN probably also plays a role in the process of iron uptake into cells. When there is no properly functioning CPN in the body, the iron oxidation process does not occur with sufficient efficiency. As a result, the amount of Fe^3+^ and Tf-Fe decreases, and the amount of NTBI and free Fe^2+^ ions increases. Subsequently, neuronal uptake of NTBI increases. Due to the lack of CPN in neurons, Fe^2+^ is not oxidized and cannot be bound to ferritin. This may result in the accumulation of Fe^2+^ inside the cell, leading to oxidative stress, generating reactive oxygen species (ROS) and ultimately cell death [125].

CPN deficiency-related astrocyte damage is probably caused by the lack of FPN, a membrane iron exporter expressed on blood–brain barrier (BBB) endothelial cells, neurons, oligodendrocytes, astrocytes, choroid plexus, and ependymal cells. CPN has been shown to be fundamentally important for the stabilization of FPN at the basement membrane surface. In a situation of CPN deficiency, FPN is not expressed properly, which causes reduced iron excretion and increases the accumulation of iron inside cells. This, in turn, may cause iron deprivation of neurons. Moreover, it cannot be ruled out that free radicals generated in iron-loaded astrocytes may cause damage to neighboring neurons. However, the fact that massive loss of astrocytes occurs in aceruloplasminemia increases the possibility of the hypothesis that neurodegeneration may be secondary to the loss of neuronal metabolic support provided by astrocytes. Moreover, astroglial cells are believed to play a neuroprotective role, which probably involves maintaining the ionic and molecular homeostasis of the CNS microenvironment. Astrocytes maintain local levels of K^+^, Ca^2+^, iron and other metals, maintain pH, and deliver glucose and metabolic substrates to neurons. Astrocytes also remove neurotransmitters such as glutamate released from synapses and other molecules that can be toxic to cells [57,61,125].

### 4.5. Glia in Iron-Induced Neuronal Degeneration: Lesson from Parkinson’s and Alzheimer’s Diseases

Excessive iron accumulation in the brain can lead to neuronal damage even when stored within ferritin. This is because iron can be liberated from ferritin binding in its ferrous state due to the acidic nature of extracellular fluid [126] and through interactions with elements like excess superoxide radicals, nitric oxide [127] and ascorbic acid [128]. Moreover, research suggests that iron overload leads to a substantial rise in the chelate-free iron pool, exceeding ferritin’s capacity for sequestration within cells [129]. Free “labile”, redox-active ferrous iron is prone to generate highly reactive hydroxyl radicals by reacting with hydrogen peroxide (H_2_O_2_) and the superoxide anion (O^2−^), both byproducts of aerobic metabolism, in the Fenton reaction eventually causing oxidative cell damage. Thus, metabolic active cells, like neurons, seem to be especially vulnerable to iron overload-induced oxidative stress. In support of this hypothesis, many in vitro [130,131] and in vivo [132,133] studies indicate that exposure of neuronal cells to iron induces a dose-dependent increase in macromolecule peroxidation leading to neuronal loss through apoptosis, autophagy, or ferroptosis. Among those, ferroptosis is an exclusively iron-dependent form of cell death characterized by lipid hydroperoxide accumulation [134]. It leads to membrane destabilization, mitochondrial dysfunction, cytoskeletal rearrangements, and impaired protein degradation [135,136,137,138]. Nuclear erythroid 2-related factor 2 (NRF2) is pivotal in the ferroptosis pathway [139], enhancing the transcription of antioxidant genes like heme oxygenase-1 (HO-1), glutathione S-transferase and superoxide dismutase-2 (SOD2) [139]. This prevents lipid hydroperoxide accumulation by countering ROS. Ferroptosis also involves GSH depletion and/or inactivation of glutathione peroxidase 4 (GPX4), a lipid peroxide scavenger [140].

Although excessive iron might be deleterious to neuronal cells, its brain accumulation associated with aging is not generally linked to pathology, suggesting that ferritin-rich glial cells are fully capable of maintaining iron homeostasis under physiological conditions. However, the cellular composition and iron levels within particular brain regions can alter during pathological conditions accompanying degeneration. For example, in PD patients, iron deposits were present in the substantia nigra, putamen, and globus pallidus [141]. The increased iron content was mainly associated with microglia and dopaminergic neurons, but not astrocytes and ferritin-positive oligodendrocytes [142]. Also, the iron level was directly correlated with microglial intensity in the SN of post-mortem PD brains [143]. In PD, the excessive extracellular release of toxic oligomers of α-synuclein, as well as neuromelanin from dying neurons, activate microglia, resulting in the subsequent secretion of pro-inflammatory cytokines such as interleukin-1β (IL-1β) and tumor necrosis factor-α (TNF-α) [144,145]. Consequently, the increased TNFα secretion exerts elevation in DMT1 level and a decrease in FPN expression in microglia, leading to extensive iron accumulation and neuronal protection against iron-induced neurotoxicity [146]. However, when the microglial iron content significantly increases, it leads to the activation of microglial nicotinamide adenine dinucleotide phosphate oxidase 2 (NOX2) and the O_2_^•−^ release, which further potentiates the neurotoxicity. Also, the magnitude of pro-inflammatory cytokine expression by microglial cells is directly regulated by intracellular iron level [147]. Moreover, the release of IL-1β and TNF-α from microglia can impact the iron metabolism of dopaminergic neurons through IRP1-mediated DMT1 upregulation and the downregulation of FPN1 expression in the ventral mesencephalon [147]. This process accounts for the intensified influx and reduced efflux of iron, ultimately culminating in an iron overload of dopaminergic neurons. Also, changes in neuronal ferritin production due to sustained IRP1 activity, or decreased ferroxidase activity of CPN could be attributable to inflammatory-induced alteration in iron homeostasis in PD [148]. Additionally, in PD, the upregulation of SEC24B, a protein-trafficking regulator in iron-loaded microglia drives microglial ferroptosis, by which neuronal death was significantly exacerbated. Taken together, this indicates that excess iron in the PD brain activates microglia, which at the beginning of the pathology, could be beneficial, leading to efficient iron scavenging, but over time this prompts the release of proinflammatory factors, thereby exacerbating iron accumulation within dopaminergic neurons.

Similar to PD, in AD large iron-ferritin accumulation was observed in glial cells associated with senile plaques, and neurofibrillary tangles [149]. Iron can prompt the production and aggregation of amyloid-β by influencing the proteolytic activation of α-secretase and β-secretase, favoring the amyloidogenic pathway, thus generating Aβ [150]. Iron might also modulate amyloid precursor protein (APP) processing, as there is a potential iron-responsive element (IRE) in the APP gene. Elevated IL-1 levels increase IRP binding to the APP 5′-UTR, reducing APP production [151]. On the other hand, it was demonstrated that Aβ-stimulated microglial cells preferentially take up non-transferrin-bound iron, and the increase in DMT1 and ferritin levels in response to Aβ was observed in those cells [151]. Despite pronounced neuroinflammation in AD brains, iron elevation is less significant compared to PD brains. In early studies on post-mortem AD cortical regions, transferrin levels were consistently lower in white matter [152], suggesting reduced iron mobility and utilization in the brain, which could contribute to neuronal degeneration and increased peroxidative damage.

In contrast to microglia, astrocytes may employ various protective mechanisms to directly regulate free iron levels within the CNS during pathology related to neurodegeneration. These include enhancing the expression of Nrf2, GSH, and catalase to counteract redox stress associated with excessive free iron [152,153]. Additionally, astrocytes can protect neurons from iron-induced damage by sequestering free iron via transient receptor potential canonical (TRPC) channels and DMT1 [154,155]. Proinflammatory cytokines like TNF-α, and IL-6 enhance DMT1 expression while reducing ferroportin 1 expression in astrocytes, leading to increased total iron uptake and storage within astrocytes [156,157]. Excess iron in the astrocytic microenvironment prompts the upregulation of ferritin, which binds and neutralizes ferrous iron, thus mitigating its oxidative stress effects [158]. Ferritin converts ferrous iron to the less reactive ferric state and stores it within its iron core, acting as an iron reservoir. Altogether, the upregulation of iron transporters and ferritin enables astrocytes to serve as iron stores, reducing free ferrous iron levels in the microenvironment and potentially averting neuronal toxicity.

### 4.6. Glial Response and Neuronal Injury in WD—MRS Studies

Proton magnetic resonance spectroscopy (1H MRS) has been applied to numerous clinical disorders, especially for neurological disorders. Neuroinflammation with astrocytes activation in brain disorders are often associated with elevated MI, and to a lesser extent elevated total creatine (Cr) and choline-containing compounds (Cho) but neuronal injury is often accompanied by lower than normal levels of NAA and Glu.

In WD patients with neurological signs the values of the ratios Cho/Cr, Glx/Cr, and Lip/Cr were statistically higher compared to the control group, while the value of the NAA/Cr coefficient was statistically lower [159]. In patients with hepatic signs the values of the mI/Cr, Cho/Cr and NAA/Cr were statistically lower compared to the control group, while Glx/Cr and Lip/Cr were higher. Presymptomatic patients had significantly reduced mI/Cr values; Cho/Cr was also reduced and Glx/Cr was elevated [159].

In patients with neurological symptoms, there would be impaired “astrocytic-neuronal cooperation” both as a result of liver damage (decrease in NAA/Cr and increase in Glx/Cr, as a result of the increase in glutamine) and the damaging effect of “free” copper ions (membrane breakdown—increase in Cho/Cr) [159]. The decrease in NAA/Cr correlated in these patients with the severity of the neurological condition [159]. Therefore, as a result of damage to the astrocytic cells, there would be a subsequent disruption of neuronal metabolism in this group, and then, as a result of copper, further destruction of neurons. There are data documenting the reversibility of the decline in NAA/Cr values, which would provide support for this theory in WD patients [160]. MRS can therefore assess both the glial response and the neuronal injury in these conditions, and may be useful for monitoring resolution of neuroinflammation during chelating WD treatments.

### 4.7. Glial Structural Changes in WD—Neuropathological Studies

Changes in the CNS in WD affect all structures of the gray and white matter, but are particularly severe in the basal nuclei (shell, caudate nucleus) and cerebellum. Histopathologically, WD belongs to the group of primary gliopathies, characterized by hypertrophy and proliferation of astrocytes with the presence of pathological forms of the glial cells. In the course of WD, in the CNS, the copper pathologically accumulates in different regions (all structures) [161], with especially higher copper levels in the basal ganglia (especially globus pallidus, putamen, caudate). The neuropathological changes in WD are observed in all structures with gray and white matter, with basal ganglia predominance. According to pathologists, WD belongs to primary gliopathy, with astrocytes’ proliferation and hypertrophy and the presence of pathological forms of astrocytes [161,162,163]. The significance of astrocytes in copper toxicosis (like WD) is very well proven, these cells have a pivotal role in brain copper homeostasis, catching and storing the copper with MTs and further releasing it to neurons and CSF, being protective cells against copper toxicosis [161,162,163]. In the course of WD, there are three forms of pathological glia cells: 1) Alzheimer type 1 and 2 cells (described by von Hösslein and Alzheimer in 1912 in neuropathological examinations of patients with Westphal–Strumpell pseudosclerosis, further known as form of WD) and Opalski cells (described by Opalski). The Alzheimer type 1 cells are pathognomonic for WD, they are labeled by GFAP, S100B protein, as well as MTs, with positive staining of cytoplasm for copper. They proliferate causing astrogliosis. The Alzheimer type 2 astrocytes (with swollen nucleus) are evocative but not specific for hepatic encephalopathy (HE) are labeled by S100B protein but not by GFAP. And finally, Opalski cells characteristic for neurological WD (absent in hepatic form) with an origin which is still in debate—being from astrocytes that are GFAP positive, MTs positive, CD68 negative, or histiocytic [84]. Additionally, there are some data according to microglia activation in WD. Their main function is control of the immune response of CNS (activated in the course of liver cirrhosis and liver failure). As this reaction is mostly seen in patients with chronic liver injury, it should be described as non-specific for WD, but specific to hepatic disorders with different etiology [162,163].

In macroscopic gross examination, the WD brain is usually soft with loss of deep and superficial white matter, slightly atrophic, with enlarged ventricles, with the main damage being located in the middle zone of putaminal nucleus. Due to copper accumulation, necrosis becomes yellow-brownish with cavitations—it reflects the brain MRI WD abnormalities (atrophy, basal ganglia signal changes, central pontine myelinolysis as well as white matter changes) [161].

### 4.8. Biomarkers of Glia Injury in WD

There is a need for objective biomarkers of WD severity, to verify the efficacy of different treatment regimens, risk of neurological deterioration (present in up to 24% neurological WD patients) as well to differentiate the natural course of the disease from drug-induced deteriorations [164,165].

The candidates are the following: neurofilament light chain (NfL)—reflecting neuroaxonal injury; tau-protein (microtubule-stabilising protein); glial fibrillary acidic protein (GFAP)—intermediate filament (forming astrocyte cytoskeleton); ubiquitin carboxy-terminal hydrolase L1 (UCH-L1) (enzyme, protein needed for axonal stability); and S100 calcium binding protein B (S100B). All these biomarkers can be measured in serum as well as CSF [165].

The Nfl, tau protein, as well as UCH-L1, mainly reflect neuronal injury [166,167,168,169,170,171], GFAP and S100B only reflect astrocyte injuries [172,173,174]. As WD is a neurodegenerative disorder, with proven neuronal necrosis mainly in the basala ganglia (neuropathological studies) the data documenting increased serum NfL (sNfL) in the course of neurological WD are very well documented [166,167,168,169,170]. All studies performed so far documented increased serum level of NfL in WD neurological patients compared to hepatic and asymptomatic. Furthermore, the serum level of NfL correlated with the severity of WD scored in UWDRS as well as in brain MRI semiquantitative scale. The data according to other candidates for neuronal biomarkers in WD—tau-protein and UCH-L1 are conflicting and need further studies [165,166,170].

As in the course of WD the pathological form of astroglia is observed (Alzheimer I and II, Opalski cells), the biomarkers reflecting the astrocytes damage in WD are also additionally investigated in WD (GFAP and S100B protein) [161]. GFAP was described in astrocytes, in intermediate filaments, forming the astrocytes’ cytoskeletons [161,165,172].

Serum GFAP level is associated with the severity of neurological diseases like head injury (correlations with the severity of neuroimaging brain injury, as well as with the Glasgow Coma Scale), as well as with the progression of multiple sclerosis (clinical and neuroradiological) [172].

S100B is a glial-specific protein which is expressed mainly by astrocytes (but only in mature astrocytes that ensheathe blood vessels and by neural/gial antigen 2 cells) as well as in melanocytes. This protein is secreted by astrocytes during head trauma and neurodegenerative disorders (as the glia biomarker), being additionally suggested as a biomarker of blood–brain barrier stability [173].

In WD so far, three studies were performed verifying the GFAP as a biomarker of neurological injury (glia involvement). The group from the United Kingdom (Shribmann, et al.), analyzing 40 WD patients (17 hepatic and 23 neurological) and 38 healthy subjects (HS), did not find any statistically significant difference between the groups (WD = 84 ng/mL; HS = 84 ng/L); also, the WD phenotype has no impact on GFAP level (neurological = 84 ng/L; hepatic = 80 ng/L) [166].

The second study performed by a group from China evaluated a larger group including 94 WD patients (74 neurological and 20 hepatic) and 25 HS [172]. WD patients with neurological phenotype had higher serum GFAP concentrations than hepatic and controls (143.8 pg/mL vs. 107.5 p/mL vs. 86.8 pg/mL; *p* < 0.01), without statistically important differences between hepatic WD and HS. The authors additionally did not find the correlations between serum GFAP and severity of WD neurological disease scored in UWDRS and brain MRI semiquantitative scale. In a logistic regression model the presence of neurological symptoms was found as an independent factor affecting serum GFAP concentrations. Analyzing the receiver operating characteristic (ROC) curves a cut-off value of 128.8 pg/mL to distinguish the WD patients with neurological and hepatic phenotypes (with a sensitivity of 80% and a specificity of 63.5%) [172].

The last study performed by a Polish group, Misztal et al., included 171 WD patients (77 hepatic and 94 neurological) and analyzed serum GFAP and S100B. The authors found that serum GFAP concentrations were elevated only in untreated WD patients with neurological presentation, which decreased during the treatment. There was no difference between hepatic and neurological groups [173].

### 4.9. Murine Models in Determining Glia Role in WD

Research utilizing murine models has revealed significant insights into neuroglial functioning. Astrocytes are crucial for several CNS functions, such as maintaining balance in glutamate, ions, and water. They also protect against oxidative and nitrosative stress, store energy, generate mitochondria, and are involved in scar formation. Additionally, astrocytes facilitate tissue repair through angiogenesis and neurogenesis, and play a significant role in modulating synapses. Yet it has become increasingly clear that there are considerable disparities between mouse and human glia [174]. These differences manifest in various aspects including developmental processes [175,176], physiological traits during maturation [177], intracellular metabolic processes [178] transcriptomic and signaling profiles [179], highlighting just a few of the fundamental discrepancies.

It is widely recognized that murine gliogenesis typically succeeds an initial phase of neurogenesis, following what is termed the “gliogenic switch” [180]. However, in humans, unlike in rodents, the processes of neurogenesis and gliogenesis occur concurrently to a large extent [181]. In terms of genetic expression, mouse astrocytes demonstrate higher levels of genes involved in mitochondrial respiration, whereas human astrocytes show an enhanced expression of genes related to the defense response, as well as those associated with the extracellular matrix and secretion. These differences are thought to contribute to the mouse astrocytes’ increased resilience to oxidative stress and to the heightened vulnerability of human cells to neurodegenerative diseases or severe injuries where oxidative damage plays a crucial role in the disease progression [182].

The copper levels in glial cells, particularly astrocytes, are considerably higher than those in neurons, indicating that astrocytes have a vital role in managing copper metabolism in the brain through interactions between glia and neurons [183]. In WD, it is probable that astrocytes accumulate substantial quantities of excess copper in the brain, thereby shielding neurons from copper toxicity [184].

In the rat brain, Atp7b was detected in the hippocampus, in the granule cells of the dentate gyrus, and in the pyramidal cells of the CA1-CA4 layers, the glomerular cell layer of the olfactory organ, in bulbs and Purkinje neurons, cerebellum, cortical pyramidal neurons, and nuclei of several nuclei in the brainstem (e.g., pontine and lateral reticular nuclei). In these brain regions, both Atp7b mRNA and protein correlated with copper distribution. Based on similar copper enzymes such as dopamine β-hydroxylase (DBH) and Cu-Zn superoxide dismutase (Cu-Zn SOD) in the Long–Evans cinnamon (LEC) rat model, authors hypothesized that ATP7B-mediated regulation of copper homeostasis in these brain regions is important in regulating DBH function [185].

ATP7B seems to play a biosynthetic role in facilitating the in-brain production of copper-dependent enzymes, including CPN. Direct copper delivery to apo-CPN in murine CNS is mediated by ATP7B [186]. However, results of Kuo’s research suggest that there can be no prenatal expression of Atp7b mRNA in the brain of the developing mouse embryo [187]. So murine model congenital glial and neuronal Cu concentration disturbances should be minimal.

In the brain, astrocytes and microglia serve as primary producers of CPN. Within these cells, holo-CPN is either secreted or bound to the membrane surface via a GPI anchor [125]. This complex (CPN-GPI) also regulates iron metabolism and its oxidation status—Fe^2+^/Fe^3+^. Additionally, several mutations in the ATP7B protein can lead to the direct accumulation of copper inside astrocytic feet [188]. According to Orre’s results, astrocytes and microglia during neurodegenerative diseases develop a more inflammatory phenotype while diminishing their neuronal support capabilities, like glutamate recycling and enhancing neuronal signaling for astrocytes, and a decrease in endocytosis for microglia. This work suggests that a persistent activation of astrocytes is associated with a decreased support for neurons, potentially contributing to the impaired neural communication that may stay behind cognitive decline during course of the disease [189].

The only original work directly describing the glial aspect of the murine model of Wilson’s disease was published by Terwel et al. They identified copper accumulation in the hippocampus of the toxic milk mouse (Rauch), with the degree of inflammatory response in various tissues appearing to correlate with the extent of copper buildup. Notably, these inflammatory changes were confined to astrocytes in the hippocampus and to microglial cells in the striatum. While the exact cause of this phenomenon remains uncertain, it is suggested that astrogliosis can sometimes be more pronounced than microgliosis. This could be linked to the syncytial way that astrocytes function. In other models of neurodegeneration, e.g., Alzheimer’s disease, astrogliosis also tends to be more extensive than microgliosis. But in the case of toxic milk mice, there is a chance that microgliosis might not reach the threshold in the hippocampus area. Additionally primary disruptions in neuronal function might change the response of astrocytes, being major support of neuronal metabolism. The mentioned regional differences in GFAP expression also suggest that GFAP metabolism is rather influenced by other factors beyond inflammation alone [190].

## 5. Summary and Conclusions

Abnormalities of copper metabolism in WD are associated with impaired iron metabolism. Both of these elements are redox active and may contribute to neuropathology. It has long been assumed that among parenchymal cells, astrocytes have the greatest impact on copper and iron homeostasis in the brain. Capillary endothelial cells are separated from the neuropil by astrocyte terminal legs, putting astrocytes in an ideal position to regulate the transport of iron and copper to other brain cells and protect them if metals breach the blood–brain barrier. Astrocytes are responsible for, among others, maintaining extracellular ion homeostasis, modulating synaptic transmission and plasticity, obtaining metabolites, and protecting the brain against oxidative stress and toxins. However, excess copper and/or iron causes an increase in the number of astrocytes and their morphological changes, as well as a loss of the copper/iron storage function leading to macromolecule peroxidation and neuronal loss through apoptosis, autophagy, or cuproptosis/ferroptosis. Understanding the mechanisms of glial involvement in neuroprotection/neurotoxicity is important for explaining the pathomechanisms of neuronal death in WD and, in the future, perhaps for developing more effective diagnostic/treatment methods.

## Data Availability

No new data were created or analyzed in this study.
